# Incremental value of SPECT/CT in detection of Meckel’s diverticulum in a 10-year-old child

**DOI:** 10.1186/s40064-016-2928-4

**Published:** 2016-08-05

**Authors:** Qian Xie, Qingjie Ma, Bin Ji, Shi Gao, Qiang Wen

**Affiliations:** Department of Nuclear Medicine, China-Japan Union Hospital, Jilin University, 126 Xiantai Avenue, Changchun, 130033 China

**Keywords:** Meckel’s diverticulum, Planar scintigraphy, SPECT/CT

## Abstract

**Introduction:**

Meckel’s diverticulum is a common congenital abnormality of gastrointestinal tract in children. Planar scintigraphy using Technetium-99m pertechnetate is widely used in the diagnosis of Meckel’s diverticulum. Single photon emission computed tomography/computed tomography (SPECT/CT) fusion imaging may help to locate the Meckel’s diverticulum lesion. We now present a Meckel’s diverticulum case which tends to be missed.

**Case description:**

The patient was diagnosed with Mecke’s diverticulum by planar scintigraphy in 2007. After seven years, a recurrence of hematochezia made the patient undergo planar scintigraphy again. However, the concentration on planar image was located at the right kidney level, we could not determine whether it was caused by physiological uptake of the right kidney or by an ectopic gastric mucosa. Using SPECT/CT technique, we confirmed that the lower part of the concentration was from a Meckel’s diverticulum from the small intestine based on the functional and anatomical information together.

**Discussion and Evaluation:**

For concentrations about the kidney level, planar scintigraphy is not enough to be diagnostic of Meckel’s diverticulum. SPECT/CT imaging may be beneficial for a definitive diagnosis. Also, fusion images may provide precise localization of the lesion. To make sure that patients obtain optimal benefit from a SPECT/CT examination, we have to balance the priority between information of anatomic location and avoiding redundant radiation to the patients.

**Conclusions:**

Our case study suggest that for cases with ambiguous planar scintigraphy images, SPECT/CT imaging should be performed to obtain a definitive diagnosis.

**Electronic supplementary material:**

The online version of this article (doi:10.1186/s40064-016-2928-4) contains supplementary material, which is available to authorized users.

## Background

Meckel’s diverticulum is a common congenital abnormality of gastrointestinal tract. The incidence is about 2–3 % worldwide (Levy and Hobbs 2004). Although bleeding, bowel obstruction, vomiting and nausea are commonly seen (Menezes et al. 2008; Ruscher et al. 2011; Cobellis et al. 2007; Mukai et al. 2002; Prasad et al. 2006; Rashid et al. 2012; Rho et al. 2013), typical symptoms and specific signs are difficult to identify. Multiple techniques have been used to detect Meckel’s diverticulum. However, the value of X-ray barium meal radiography and video image endoscope in detecting Meckel’s diverticulum is limited, because some patients cannot tolerate discomfort or pain during these examination processes. More than 90 % of Meckel’s diverticulum with gastrointestinal bleeding is with heterotypic gastric mucosa. As ectopic gastric mucosa could take in and concentrate Technetium-99 m pertechnetate promptly, planar scintigraphy has been widely used in the detection of Meckel’s diverticulum. Hybrid single photon emission computed tomography/computed tomography (SPECT/CT) enables a direct correlation of anatomical information and functional information, resulting in better localization and definition of scintigraphic findings (Even-Sapir et al. 2009). It is now more and more frequently used in the diagnosis of Meckel’s diverticulum.

## Case report

**Fig. 1:**
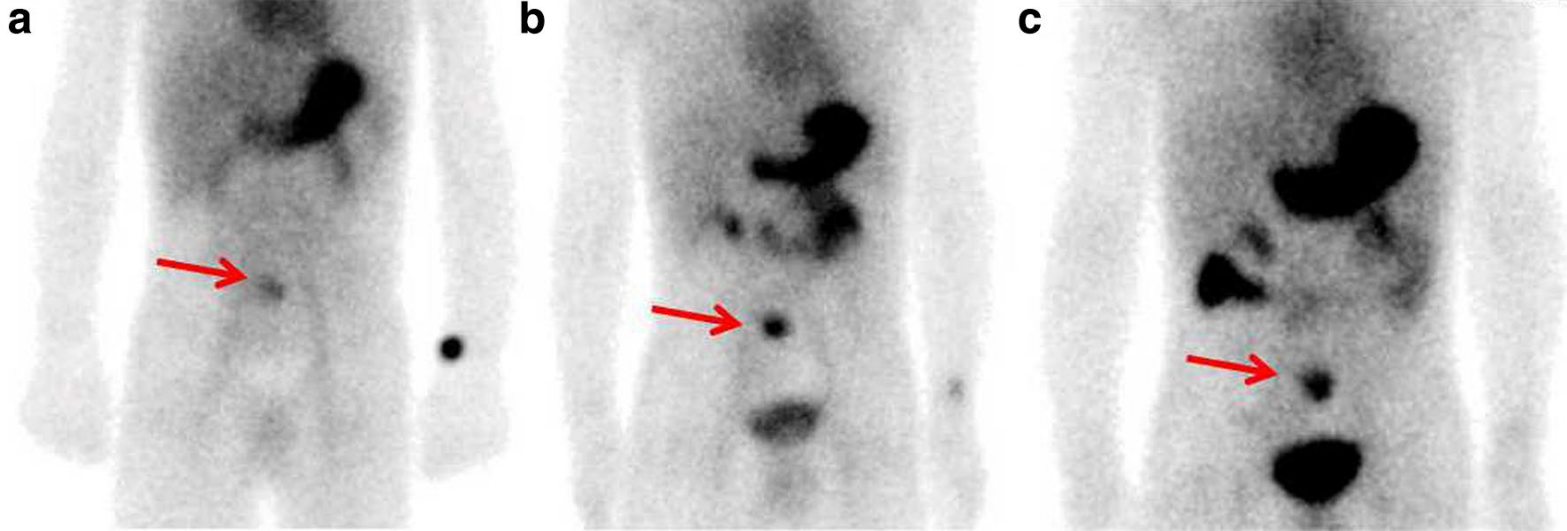
5 min (**a**), 20 min (**b**) and 60 min (**c**) after administering 1 mCi of Technetium-99 m pertechnetate intravenously, planar scintigraphy was performed respectively. Images showed a focal concentration at the small intestine region of the lower abdomen

A 3-year-old female patient was admitted in our institution due to 3 days of maroon stools in 2007. No active bleeding was found by gastroscopy, colonoscopy or abdominal/pelvic CT. Planar scintigraphy showed a focal concentration at the small intestine region of the lower abdomen (Fig. 1). She was diagnosed as Meckel’s diverticulum. Because this patient has a history of congenital heart disease, she did not have a surgery with this lesion. She stopped having maroon stools the following day and left the hospital.

**Fig. 2:**
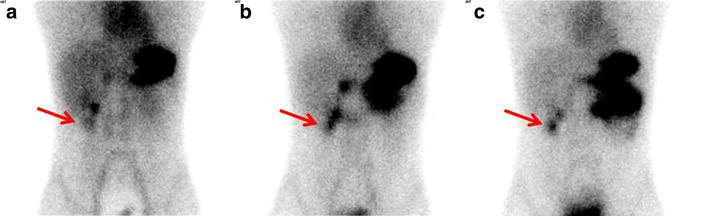
5 min (**a**), 20 min (**b**) and 60 min (**c**) after administering 1.5 mCi of Technetium-99 m pertechnetate intravenously, planar scintigraphy was performed respectively. Images showed two focal concentrations under the liver at the right lower quadrant of abdomen region

The patient was again admitted in our hospital due to massive hematochezia in 2013. Physical examination was normal except for an abdominal tenderness. Serum hemoglobin concentration was 76 g/L. Esophagogastroscopy (EGD), colonoscopy and abdominal/pelvic CT were done and no active bleeding site was found. To look for the source of bleeding, we performed a planar scintigraphy again. We found two focal concentrations under the liver at the right lower quadrant of abdomen region. We could not determine whether it was caused by physiological uptake of the right kidney or by an ectopic gastric mucosa (Fig. 2). Delayed imaging was performed after 20 and 60 min, respectively. At the delayed phase, the degree of the uptake in the lower part of this concentration changes simultaneously with that of the stomach.

**Fig. 3:**
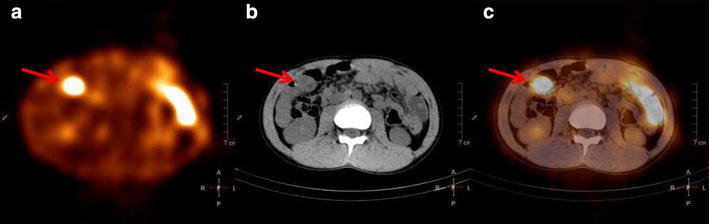
Axial SPECT/CT image of the Meckel’s diverticulum. **a** Axial image SPECT shows one focal concentration in the right abdomen (*arrow*). **b** CT demonstrated a blind-ending tubular structure in the right abdomen (*arrow*). Anatomical relationship between the focal concentration and right kidney cannot be identified. **c** Axial SPECT/CT fusion image showed that it was in front of the kidney that the focal concentration lays (*arrows*)

Considering that the patient had been diagnosed with Meckel’s diverticulum 7 years ago, we performed a SPECT/CT fusion imaging to the patient (Fig. 3). Based on the fused image, we found that the focal concentration was not from the right kidney. Therefore, a diagnosis of Meckel’s diverticulum was confirmed. The patient underwent surgical diverticulectomy the following day. The surgeon found a 3 cm Meckel’s diverticulum which located in the ileum within 15 cm of the ileocecal valve. Histological examination of the lesion revealed an ectopic gastric mucosa. The patient remains asymptomatic without any gastrointestinal bleeding or maroon stools 14 months after the surgery.

## Discussion

Technetium-99 m pertechnetate planar scintigraphy is widely used in the diagnosis of Meckel’s diverticulum (Sfakianakis and Conway 1981). Because Technetium-99 m pertechnetate is excreted by the urinary system, kidney and urinary tract are normally with concentrations, which may overlap with that in Meckel’s diverticulum on the planar image and lead to false result. In our case, a diagnosis of Meckel’s diverticulum has been made by a former planar scintigraphy. However, when the patient underwent planar scintigraphy 7 years later, we were unable to determine whether the focal concentration was caused by physiological uptake of the right kidney or by ectopic gastric mucosa. To look for the exact anatomic site of the focal concentration, we performed a SPECT/CT scan to the patient. The educational significance of our report was that, for the Meckel’s diverticulum of the kidney level, SPECT/CT should be always performed to avoid false result due to physiological uptake of the kidney. Due to the puberty age of the patient, the relative position of Meckel’s diverticulum may have been changed compared to the lesion identified 7 years ago.

The use of SPECT/CT fusion imaging provided valuable diagnostic information and avoided false negative study (Swaniker et al. 1999; Jonathan 2009). Once a Meckel’s diverticulum was missed, the patient would not receive effective treatment and suffer serious further complications. Furthermore, SPECT/CT fusion imaging may provide functional and anatomical information together to help surgeons to plan the surgery.

Compared with planar scintigraphy, SPECT/CT is with relatively high cost and increased radiation from the CT portion. Also, Meckel’s diverticulum patients are usually children under 10 years old. Therefore, SPECT/CT fusion imaging still has not been routinely used in diagnosis of Meckel’s diverticulum. In our case, to minimize radiation exposure to the patient, we set the scanning range from 2 cm below the xiphoid to inferior pole of kidneys to locate the lesion. The tube voltage was set at 100 kV and the tube current was set at 20 mA (Gelfand and Lemen 2007; Lee et al. 2015; Goo 2012). Through these methods, the radiation dose of the CT portion can be reduced by more than 60 %.

For concentrations about the kidney level, planar scintigraphy is not enough to be diagnostic of Meckel’s diverticulum. SPECT/CT imaging may be beneficial for a definitive diagnosis. Also, fusion images may provide precise localization of the lesion. To make sure that patients obtain optimal benefit from a SPECT/CT examination, we have to balance the priority between information of anatomic location and avoiding redundant radiation to the patients.

## Conclusion

For cases with ambiguous planar scintigraphy images, SPECT/CT imaging should be performed to obtain a definitive diagnosis.

## Additional file

10.1186/s40064-016-2928-4 Information about the patient.
