# Supplementary material for: Incremental value of SPECT/CT in detection of Meckel’s diverticulum in a 10-year-old child
Source: Springerplus. 2016 Aug 5;5(1):1270. doi: 10.1186/s40064-016-2928-4 (PMC4975725; doi:10.1186/s40064-016-2928-4)
Supplement: Supplementary file 1 — 10.1186/s40064-016-2928-4 Information about the patient. [file 40064_2016_2928_MOESM1_ESM.doc]

**Information about the patient**

Information about the patient that could make her identifiable

 Age: 12-year-old;

Gender: Female;

Hospital where he was admitted: China-Japan Union Hospital;

Condition: The patient was admitted to our institution due to a 3-day history of maroon stools in 2007 and she was diagnosed as MD by SPECT. She was again admitted by our hospital due to massive hematochezia in 2013, we confirmed that she was MD positive by SPECT/CT.

I confirm that I have read and understood the terms and conditions of the ethical guidelines as set out by COPE. And I confirm that my submission meets the ethical guidelines, including adherence to the legal requirments of the study country. I confirm that my manuscript has not been assessed scientifically yet.
